# Outcome of coronary plaque burden: a 10-year follow-up of aggressive medical management

**DOI:** 10.1186/1476-7120-8-5

**Published:** 2010-03-12

**Authors:** Victor K Goh, Chu-Pak Lau, Stefan Mohlenkamp, John A Rumberger, Stephan Achenbach, Matthew J Budoff

**Affiliations:** 1 Imaging Centre, Matilda International Hospital, 41 Mount Kellett Road, The Peak, Hong Kong, SAR China; 2 Consultant Cardiologist, Hong Kong Sanatorium and Hospital, 2 Village Road, Happy Valley, Hong Kong, SAR China; 3 Department of Cardiology, West German Heart Center Essen, University Clinic Essen, Hufelandstrasse 55, 45122 Essen, Germany; 4 Department of Cardiac Imaging, Princeton Longevity Center, Princeton Forrestal Village, 136 Main Street, Princeton, NJ 08540, USA; 5 Department of Internal Medicine 2, University of Erlangen, Ulmenweg 18, 91054 Erlangen, Germany; 6 Department of Internal Medicine, Division of Cardiology, Los Angeles Biomedical Research Institute at Harbor-UCLA, 1124 West Carson Street, Torrance, CA 90502, USA

## Abstract

**Background:**

The effect of aggressive medical therapy on quantitative coronary plaque burden is not generally known, especially in ethnic Chinese.

**Aims:**

We reasoned that Cardiac CT could conveniently quantify early coronary atherosclerosis in our patient population, and hypothesized that serial observation could differentiate the efficacy of aggressive medical therapy regarding progression and regression of the atherosclerotic process, as well as evaluating the additional impact of life-style modification and the relative effects of the application of statin therapy.

**Methods:**

We employed a standardized Cardiac CT protocol to serially scan 113 westernized Hong Kong Chinese individuals (64 men and 49 women) with Chest Pain and positive coronary risk factors. In all cases included for this serial investigation, subsequent evaluation showed no significantly-obstructive coronary disease by functional studies and angiography. After stringent risk factor modification, including aggressive statin therapy to achieve LDL-cholesterol lowering conforming to N.C.E.P. ATP III guidelines, serial CT scans were performed 1-12 years apart for changes in coronary artery calcification (CAC), using the Agatston Score (AS) for quantification.

**Results:**

At baseline, the mean AS was 1413.6 for males (mean age 54.4 years) and 2293.3 for females (mean age 62.4 years). The average increase of AS in the entire study population was 24% per year, contrasting with 16.4% per year on strict risk factor modification plus statin therapy, as opposed to 33.2% per year for historical control patients (p < 0.001). Additionally, 20.4% of the 113 patients demonstrated decreasing calcium scores. Medical therapy also yielded a remarkably low adverse event rate during the follow-up period --- 2 deaths, 2 strokes and only 1 case requiring PCI.

**Conclusions:**

This study revealed that aggressive medical therapy can positively influence coronary plaque aiding in serial regression of calcium scores.

## Background

It is not generally known what happens to the coronary calcium score when patients are subjected to aggressive medical therapy on long-term follow-up. Furthermore, there is a paucity of information on the prevalence of coronary artery disease in some groups of individuals, especially certain ethnic groups such as the Chinese. Although there are data available on Chinese patients [1-5], such data usually touch on a few selected parameters and rarely focus on the prevalence aspect of coronary artery disease in the Chinese.

After CT cardiac imaging of the coronary system became established as a means of detecting underlying atherosclerosis [6], serial scans were performed in some studies to follow progress of disease in certain special groups of individuals [7,8].

The MESA study [9] observed that lipid levels were related to baseline coronary artery calcification (CAC) burden but not to CAC progression. Similarly, DA Anand et al. [10] found that statins were unable to prevent CAC progression. On the other hand, P Raggi et al. [11] found that CAC progression in diabetic subjects was greater in the absence of statins. ND Wong et al. [12] then opined that only HDL Cholesterol rather than LDL Cholesterol was related to CAC progression.

Past and present uncertainties on the causes of CAC progression are a natural impetus for further research into this subject. Our thoughts on this area led us to reason that Cardiac CT could be applied effectively to quantify the burden of coronary atherosclerosis, with the hypothesis that performing serial scans could shed light on the efficacy of aggressive medical therapy and hence improve management of CAD, particularly in assessing possible factors that govern progression and regression of the atherosclerotic process, secondly in determining any difference between giving statins alone and administering statins together with life-style modification, and thirdly in evaluating whether type and dose of statins would make a difference. Furthermore, since the development of atherosclerotic plaque literally takes decades, a longer term follow-up than had been applied in prior studies was felt to provide a better comprehension of long-term consequences to therapeutic interventions.

113 patients with non-critical coronary disease constituted the population for this long-term follow-up study. This group, which comprised 64 men and 49 women, had two or more consecutive CT coronary calcium scans at least 12 months apart. Their response to aggressive non-invasive, medical therapy is described below.

## Methods

### Patient Demographics and Entry Criteria

**Figure 1:**
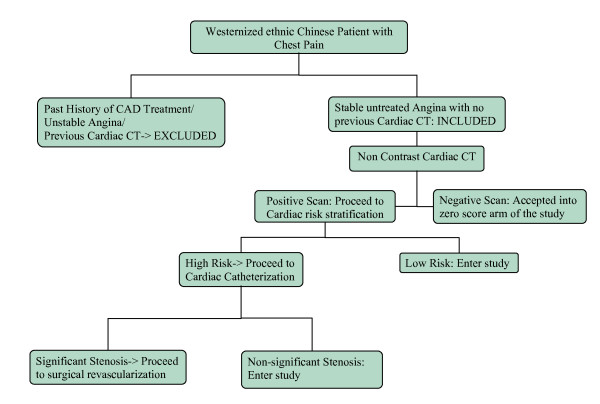
**Flow Chart showing how patients were selected for entering the study**.

We studied a group of westernized ethnic Chinese urban inhabitants with positive risk factors and chest pain. At entry, patients were interviewed and gave informed consent. Those who had been undergoing a previous course of treatment for coronary artery disease were excluded, as were patients who had unstable chest symptoms. Only those presenting with chest symptoms for the first time and who had never had a prior cardiac CT examination were accepted into the study. They firstly received a non-contrast CT scan for calcified coronary artery plaque determination. Cases with a positive scan then underwent traditional testing for stratification of cardiac risk. Those patients found to be at high risk and who had significant coronary stenosis on subsequent coronary angiography proceeded to revascularization and were not included in this investigation. The remaining subjects were either free of coronary atheromatous plaque or had non-critical coronary artery disease. These patients were then selected for the study. This group eventually comprised 64 men aged between 28 and 83 years (mean age 54.4 years) and 49 women aged between 36 and 86 years (mean age 62.4 years) [Figure 1].

The estimated pretest likelihood of significant coronary artery disease [13] of this study group is given in Table 1 (< 50% likelihood in 32 out of 113 patients (= 28.3%), 50 - 80% likelihood in 37 out of 113 patients (= 32.7%), and > 80% likelihood in 26 out of 113 patients (= 23%)).

**Table 1:** Cases presenting with Chest Pain to compute pretest likelihood of CAD

	Atypical Chest Pain		Typical Chest Pain	
**Age**	**Men**	**Women**	**Men**	**Women**

20 - 29	1 case	0	0	0

30 - 39	21.8% × 5 cases	4.2% × 1 case	69.7% × 1 case	25.8% × 0

40 - 49	46.1% × 13 cases	13.3% × 2 cases	87.3% × 8 cases	55.2% × 0

50 - 59	58.9% × 10 cases	32.4% × 11 cases	92.0% × 6 cases	79.4% × 5 cases

60 - 69	67.1% × 5 cases	54.4% × 11 cases	94.3% × 5 cases	90.6% × 7 cases

70 - 79	5 cases	6 cases	4 cases	5 cases

80 - 89	1 case	1 case	0	0

Overall	< 50% Likelihood: 32/113 (= 28.3% of cases)	50 -- 80% Likelihood: 37/113 (= 32.7% of cases)	> 80% Likelihood: 26/113 (= 23% of cases)	

### Protocol

All patients with chest pain and positive coronary risk factors meeting the above-mentioned criteria for inclusion received a blood test to determine their biochemical Risk Profile (including Total Cholesterol, HDL-Cholesterol, LDL-Cholesterol, VLDL-Cholesterol, Triglycerides, Glucose, Uric Acid). As mentioned, they also had a non-contrast CT of their coronary system. Patients who had positive calcium scores of 400 Agatston score or above (indicating severe disease) then underwent a stress test for stratification of cardiac risk. Eventually 20 patients with a heavy calcium score and a positive stress test were catheterized for their intraluminal status. 19 patients with significant coronary stenosis on coronary angiography proceeded to revascularization and were excluded. The 113 remaining risk factor-bearing symptomatic patients (with either no coronary plaque burden or having non-critical coronary disease) then constituted the subjects for this long-term follow-up study.

Patients started the study in 1990-1992, and were rescanned as late as 2002, with the time between scans averaging 10 ± 1.5 years.

### Instrumentation

All patients underwent electron beam Computerized Tomography of their coronary arteries by a standard protocol [14]. The cardiac CT studies (both at baseline and follow-up) were performed with an Imatron C-100 and subsequently a C-150 electron beam computed tomographic scanner (Imatron, South San Francisco, California) in high-resolution volume mode using a 100-ms exposure time as previously described [15]. As the study progressed, it became apparent that electrocardiographic triggering was desirable to enable each image to be obtained during the quiet time of the cardiac cycle, which eventually corresponded to 40% of the RR interval [15,16], while scans were initially non-gated during the early 5 years of the study (comprising about one-third of the total patient population). Proximal coronary artery visualization was obtained, and 30 or more consecutive images were obtained at 3-mm intervals. Total radiation exposure using this technique was <1 mSv per participant per session (which low value was achievable because of the extremely rapid prospective acquisition of data by EBCT, and which is superior even to later generations of MDCT scanners by not involving any mechanically-movable parts in its hardware that could slow down scanner activity to require multiple sequences with protracted irradiation). Participants were included in this study only if complete data were available from the scans, without mis-registration of slices due to motion artifacts, respiration, or poor electrocardiographic triggering.

### Image Analysis

The site and extent of calcification of each diseased vessel was documented and stored for future re-analysis/comparison. In the acquired images, calcification was defined as a plaque of at least 3 contiguous pixels (area 1.03 mm^2^) with a density of >130 Hounsfield units. The amount of calcium was quantified using a lesion score. This score, as described by Agatston et al. [14], was calculated by multiplying the lesion area by a coefficient based on the peak density within that plaque. The total Agatston Score of the participant was determined by summing the scores obtained for each lesion. The voxel size on cardiac CT scans obtained with a 3-mm section thickness and a typical field of view of 30 cm^2 ^(pixel size = 0.586 mm) corresponds to 0.586 × 0.586 × 3 = 1.03 mm^3^. The original voxel was divided into smaller voxels each of whose size was 0.201 mm^3 ^with the technique of isotropic interpolation for measuring a more precise volume [17,18]. All voxels with a value greater than 130 Hounsfield units were defined as a calcified lesion. A total coronary artery score by the Agatston method was determined by summing individual lesion scores from each of 4 anatomic sites (left main, left anterior descending, circumflex, and right coronary arteries). A single experienced investigator, blinded to clinical status of the participant and temporal relation of the scans, interpreted all studies on a commercially available software package (AccuImage Diagnostics Corp., South San Francisco, California).

Intra- and Inter-observer variability in CT calcium scoring has been reported [19]. Since only one investigator read all the scans, this eliminated inter-observer variability. Intra-observer variability has been found to be low [19], the correlation being on the order of 0.99.

### Treatment

The number of risk factors for a participant was calculated based on the National Cholesterol Education Program guidelines [20-22]. Risk Factors included: current cigarette smoking, history of premature coronary disease in a first-degree relative, diabetes mellitus, hypertension, and hypercholesterolemia. Cigarette smoking was defined as use of >10 cigarettes/day. Participants using insulin or oral hypoglycemic agents were classified as diabetic. Hypertension was defined by use of antihypertensive medication or known and untreated hypertension, and hypercholesterolemia was similarly defined by use of cholesterol-lowering medication or known but untreated high cholesterol (total>6.3 mmol/L).

When calcified plaques were demonstrated, individuals would be counseled about possible dangers (such as development of coronary events), and advised on life-style modification. Typically patients would be advised to start a low cholesterol diet and asked to do slow-paced exercises for at least 1 hour each day, according to N.C.E.P. guidelines, starting with the ATP I, proceeding on to the ATP II and finally the ATP III guidelines when each new set of guidelines became available [20-22]. Drug therapy for hypercholesterolemia was only started after obtaining lipid and body-organ biochemistry as baseline parameters to ensure tolerability to drug treatment. Statins were only started when patients met the criteria for such treatment with respect to ATP guidelines, while calcium scores were not relied upon in committing patients to statin therapy. The dosage of statins used started from the lowest recommended dose for each choice of statin and was individualized for each patient to enable him/her to achieve LDL cholesterol levels as per the NCEP guidelines operating at the time. The maximal dose of each choice of statin is listed in Table 2. Subsequently, patients were asked to return for regular clinical follow-up by one of the authors as well as to report any new developments even before their next due appointment.

**Table 2:** (Comparison of Progressors and Regressors in Calcium Scores)

PARAMETERS	Patients showing Progression of Calcium score	Patients showing Regression of Calcium score
Age as a risk factor	53/70 subjects (= 75.7%)	19/23 subjects (= 82.6%)

Positive Family History	28/70 subjects (= 40%)	10/23 subjects (= 43.5%)

Hypertension	37/70 subjects (= 52.9%)	13/23 subjects (= 56.5%)

Low HDL	23/70 subjects (= 32.9%)	9/23 subjects (= 39.1%)

Smoking	16/70 subjects (= 22.9%)	2/23 subjects (= 8.7%)

Diabetes mellitus	17/70 subjects (= 24.3%)	6/23 subjects (= 26.1%)

Unhealthy Diet	63/70 subjects (= 90%)	19/23 subjects(= 82.6%)

Lack of Exercise	13/70 subjects (= 18.6%)	8/23 subjects (= 34.8%)

Mental Stress	15/70 subjects (= 21.4%)	5/23 subjects (= 21.7%)

Lipid Lowering Treatment (Maximum Daily Dosage)	Atorvastatin 120 mg	Atorvastatin 180 mg
	Simvastatin 40 mg	Simvastatin 60 mg
	Lovastatin 120 mg	Lovastatin 80 mg
	Pravastatin 40 mg	Pravastatin 40 mg
	Fluvastatin 20 mg	Fluvastatin Absent
	Gemfibrozil 1,800 mg	Gemfibrozil 900 mg
	Fenofibrate 300 mg	Fenofibrate Absent
	Nicotinic Acid 200 mg	Nicotinic Acid Absent
	Benfluorex 450 mg	Benfluorex 900 mg
	Omega-3 Absent	Omega-3 tab 1
	Sibutramine Absent	Sibutramine 10 mg
	Cholestyramine 4 grams	Cholestyramine 8 grams
	Ezetimibe 10 mg	Ezetimibe 10 mg

Other Medications	AB (Alpha Blockers)	AB Absent
	ACEI (ACE Inhibitors)	ACEI (ACE Inhibitors)
	AMIO (Amiodarone)	AMIO Absent
	APR (Allopurinol)	APR (Allopurinol)
	ARB (Angiotensin Receptor Blocker)	ARB (Angiotensin Receptor Blocker)
	ASP (Aspirn)	ASP (Aspirn)
	AZL Absent	AZL (Alprazolam)
	BB (Beta Blockers)	BB (Beta Blockers)
	CCB (Ca. Channel Blockers)	CCB (Ca. Channel Blockers)
	CLP (Clopidogrel)	CLP (Clopidogrel)
	DIG (Digoxin)	DIG Absent
	DR (Diuretics)	DR (Diuretics)
	GTN Absent	GTN (Glyceryl Trinitrate)
	INS (Insulin)	INS Absent
	ISD (Isosorbide Dinitrate)	ISD Absent
	ISM (Isosorbide Mononitrate)	ISM (Isosorbide Mononitrate)
	OH (Oral Hypoglycaemics)	OH (Oral Hypoglycaemics)
	POE (Plant Oestrogens)	POE (Plant Oestrogens)
	PPF Absent	PPF (Propafenone)
	T3 (Triiodothyronine)	T3 Absent
	T4 (Thyroxine)	T4 (Thyroxine)
	TM (Trimetazidine)	TM (Trimetazidine)
	WAR (Warfarin)	WAR Absent

### Statistical analysis

With each subsequent review, patients' history and clinical/laboratory findings were recorded. In particular, each patient's life-style status was re-assessed by going though a check-list of parameters to track patients' progress. The degree of progression was calculated as the annualized percent difference between the 2 scans, and comparisons of mean relative changes in the calcium score were made with the student's T test. A rising trend in the serial calcium score was defined as either progressive elevation in score value or at least a rise in score between two consecutive scans, while a decreasing trend was the opposite. A 2-sample z test was used to document the significance of the difference between groups in the proportion of participants with any given risk factor or intervention. Hypertension, tobacco use, diabetes, age, gender, family history of premature heart disease, number of risk factors, hypercholesterolemia and the utilization of statin therapy were evaluated as independent variables.

## Results

### Demographics/Calcium Score Range (at study entry)

Tables 3 and 4 show the demographics and range of calcium scores of subjects in the study at baseline entry. The mean calcium score was 1413.6 for Males and 2293.3 for Females (females being nearly a decade older than males).

**Table 3:** Patient Characteristics at study entry

	Number of Patients	Age Range	Mean Age	Calcium Score Range	Mean Calcium Score
					

Male	64	28 - 83	54.4	0 - 2827.1	1413.6

					

Female	49	36 - 86	62.4	0 - 4586.6	2293.3

					

Entire Field	113	28 - 86	57.9	0 - 4586.6	2293.3

(Demographics and Range of Calcium Scores of the entire study group)

**Table 4:** Calcium Scores according to Age

Age	Men	Mean Score	Women	Mean Score
20 - 29	0 - 1	0.5	0	0

30 - 39	0 - 489.5	244.75	0	0

40 - 49	0 - 1774.3	887.2	0 - 5.5	2.8

50 - 59	0 - 657	328.5	0 - 195	97.5

60 - 69	0 - 1715.8	857.9	0 - 587.3	293.7

70 - 79	0 - 1612.5	806.3	0 - 849.7	424.9

80 - 89	967.8 - 2827.1	1897.5	0.52 - 4586.6	2293

### Progress of Calcium Score Results

All patients were consistently kept on aggressive statin therapy to keep their LDL-Cholesterol level according to N.C.E.P. guidelines operative at the time (<100 mg/dl or <2.63 mmol/L). All medical problems (e.g. hypertension, diabetes) were checked to ensure persistent optimal control (optimal blood pressure was defined as < 120-130/70-80; optimal fasting blood sugar was defined as < 6.1 mmol/L). Finally, all patients were interviewed at each clinical follow-up session regarding the completeness of their life-style modification, with cross-referencing made with family members to ascertain accuracy. The study revealed the progress of patients' calcium scores proceeded along several different courses.

### Progression of CAC scores

**Figure 2:**
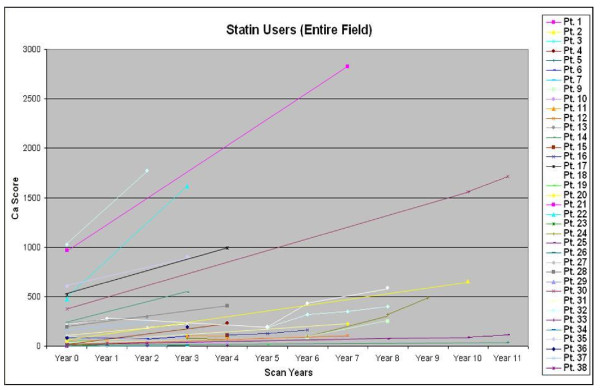
**This figure is typical of the Rate of Progression of Calcium Scores on Statin Usage**. Statins produced a retarded Rising Trend in Calcium Scores compared to historical untreated Subjects. Ca = Calcium; Pt. = Patient

A RISING TREND [Figure 2] was seen in 70 patients whether they were on stand-alone statin therapy or on Life-style Modification with statin therapy. Among these 70 patients, the average increase of calcium scores was 24%/year for 52 patients on Statin treatment alone. This contrasted with 16.4%/year for another 18 patients who were confirmed by individual specific questioning to have maintained stringent life-style modification (including a strict diet, smoking cessation and stress reduction (if applicable), regular exercise, weight control) plus aggressive statin administration, as opposed to an average of 33.2%/year for untreated patients (p < 0.001) in a previously published series [7].

### Non-progression of CAC scores

#### A) Patients starting with a baseline score of Zero

A total of 20 patients had a baseline calcium score of 0 (i.e. absence of detectable Calcium). Of these 20 patients, one group of 7 patients started with a zero score and remained at zero on serial scanning despite being on no lipid-lowering therapy.

Another group of 13 of these 20 patients also remained at a zero score, but were on treatment because of meeting criteria for drug therapy according to ATP guidelines. 8 of these 13 patients were on statin treatment alone, while another 5 subjects maintained their zero score by statin therapy plus life-style modification.

Yet another group of 14 patients started with a zero score but subsequently developed positive calcium scores on serial scanning.

#### B) Patients Starting with a baseline Positive Score Showing a Decreasing Trend

**Figure 3:**
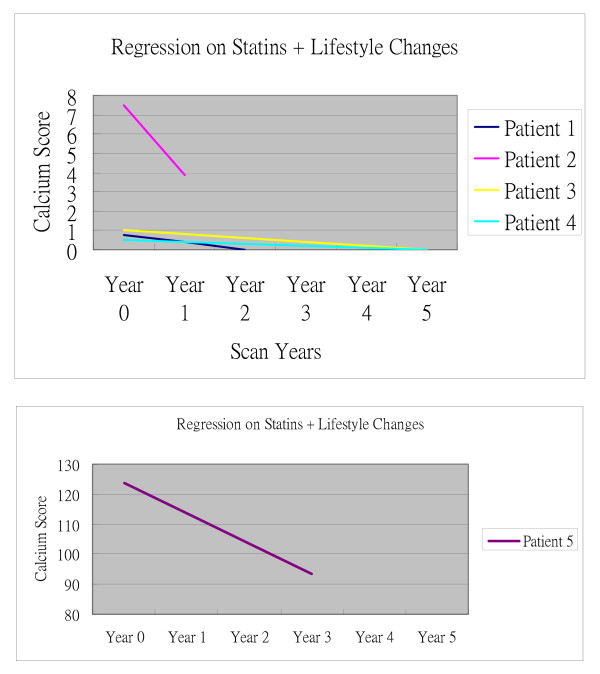
**This figure is typical of the Rate of Regression of Calcium Scores on Statin usage plus Risk Factor Modification**. Statins plus Life-style Modification regressed Coronary Plaques in some cases.

Another group of 23 patients with an initially positive calcium score had progressive improvement, with a continual reduction in calcium score values. Such a REDUCING TREND was observed in patients on stand-alone statin therapy (for 18 patients) or on life-style modification plus statins (for 5 patients) [Figure 3]. Overall, 20.4% of the 113 cases demonstrated regression in their calcium scores.

The number of patients who manifested regression in calcium score was 23 in all. This small group of patients was nevertheless unique in the respect of being able to develop a progressively reducing calcium score. Their characteristics as compared to patients who progressed in calcium score are outlined in Table 2. As can be seen, they do not display any peculiar features as compared with the other subjects in the study, and the reasons for their regression are therefore not apparent.

##### Detailed Outcomes

Of 113 subjects, 96 (85%) stayed on statins over their entire follow-up period, and maintained their cholesterol treatment goals throughout. 91 out of the 113 subjects (i.e. 80.5%) adopted a healthy diet and 90 out of the 113 subjects (= 79.6%) avoided smoking, while 81 out of 113 (i.e. 71.7%) engaged in regular exercise, and 70 out of 113 patients (i.e. 61.9%) reduced life stress. Although these individuals adapted to various life-style modifications, at the end of the study only 31 (i.e. 27.4%) persevered with these changes throughout their follow-up period.

Adverse events, over 12 years of follow-up included 2 strokes, 1 percutaneous coronary intervention and 2 deaths (1 from cancer, and 1 from suicide).

##### Possible Pointers to means of achieving differential outcomes

Analysis of patients who progressed versus those who regressed in calcified plaque burden did not reveal any specific characteristic trend in terms of age, sex, risk factor profile (except possibly for smoking) or degree of lipid-lowering.

Similarly, amongst those subjects who responded with a retarded rate of rise of calcified plaque on treatment, there was no clear-cut mechanism for this diminution in progression, as analysis of the serial lipid profiles of these patients revealed that Risk Factor Modification did not improve lipid profile as a means of achieving a lower annual rise in calcium score [Table 5].

**Table 5:** (Comparison of influence of therapy on the 3 categories of patients)

PARAMETER	Patients untreated for Hypercholesterolaemia	Patients on Statins alone		Patients on Statins + Risk Factor Modification (RFM)	
Change in Total Cholesterol after intervention	Nil	Nil		Nil	

Change in HDL Cholesterol after intervention	Nil	Nil		Nil	

Change in LDL Cholesterol after intervention	Nil	Nil		Nil	

Change in Triglycerides after intervention	Nil	Nil		Nil	

Change in Calcium score with intervention	Nil	52/70 patients (= 74.3%) progressed in Calcium score on Statins alone 18/23 patients (= 78.3%) regressed in Calcium score on Statins		18/70 patients (= 25.7%) progressed in calcium score on Statins + RFM 5/23 patients (= 21.7%) regressed in calcium score on Statins + RFM	

PARAMETERS	Patients untreated for Hypercholesterolaemia	Rising Ca score on statins alone	Decreasing Ca score on statins alone	Rising Ca score on statins + RFM	Decreasing Ca score on statins + RFM

No. of Patients	7	52 out of 70 patients (= 74.3%)	18 out of 23 patients (= 78.3%)	18 out of 70 patients (= 25.7%)	5 out of 23 patients (= 21.7%)

Calcium score	Zero at baseline and zero on follow-up	From a mean calcium score of 111.7 to 301.2 (+169.7%)	From a mean calcium score of 276.2 to 194.9 (-29.4%)	From a mean calcium score of 89.17 to 147.6 (+65.5%)	From a mean calcium score of 26.7 to 19.5 (-27%)

Furthermore, response to therapy was not related to the type of statins, but dosing was probably important since adherence to treatment goals to keep to current N.C.E.P. guidelines meant statin dosage had to be adjusted upwards to maintain satisfactory lipid levels as stipulated by the guidelines in force at the time.

## Discussion

The traditional approach to compute coronary risk has been the application of the Framingham score. However, it has been found that this scoring system may overestimate the actual risk for various populations outside the United States [23-28]. Accordingly, refinement of the actual risk has been proposed by use of various scoring techniques that apply to individual population groups. An even simpler, and possibly more accurate, means of stratifying risk is to directly visualize the coronary arteries for the presence of atheromatous plaques [29]. Cardiac CT has been used to non-invasively track changes of coronary atherosclerosis by periodically quantifying Coronary Artery Calcium (CAC) [30] and thereby demonstrating the effects of therapy on this process. Several studies have been conducted to evaluate the progression of CAC over time. Janowitz et al. [30], demonstrated that the score progression was more marked in patients with obstructive CAD compared with patients who had no clinically manifest disease (48% versus 27% during the mean 406 days of follow-up). One study, (n = 81), demonstrated an increase in the CAC score by 24% each year since baseline [31] with no specific morbidity/mortality mentioned. Mitchell et al. [32], followed 347 patients for 1.4 years, demonstrating an annual average increase in CAC scores of 21% in men and 18% in women. A small study of young patients with end-stage renal disease demonstrated a mean calcium score rise of 59% per year, with a doubling of score by 20 months [33].

Of the 113 patients we studied, all maintained their intake of statins and a number of them persevered with life-style changes. We were thus able to observe the temporal development of these risk factor-bearing symptomatic CAD cases on aggressive non-invasive therapy over a decade or more of follow-up. Increased adherence has been demonstrated with patients who had visualization of their CAC [34]. This may have explained the long term maintenance of therapies.

We demonstrated that the rate of change of CAC, as a surrogate for coronary atherosclerosis, is 16.4 to 24% per annum for a variety of underlying risk factors. The rate of change in the CAC score was slowed by the utilization of statin therapy for hypercholesterolemia, and 20.4% of participants actually exhibited outright regression in this cohort. Accurate and non-invasive measures of atherosclerosis in high-risk persons can better assess processes associated with disease progression, as well as evaluating therapies that dually prevent the progression or even induce regression of atherosclerosis/clinical CAD [35]. For this purpose, a tool is needed to assess efficacy of different interventions over relatively short time periods. Cardiac CT as a non-invasive method to measure the quantity of calcified coronary plaque, and capable of measuring rates of change over time, appeared to fulfill this role in this study.

Furthermore, cardiac CT scanning, being a non-invasive modality, proved to be a convenient means to quantify disease. By displaying a visual image that was understandable to patients it presented their condition in a way to which they could relate. This made it easier for them to keep up with their treatment and apparently motivated them to return for follow-up [34].

The findings of Achenbach et al. [36] indicated that reduction of LDL cholesterol to appropriate levels by efficacious statin therapy can significantly reduce coronary artery calcium progression, and the results of the COURAGE Trial [37] further confirmed that optimal medical therapy can be as effective as percutaneous coronary intervention in stable CAD. In this study, we were able also to observe the relative contribution of life-style adjustments versus drug therapy in terms of efficacy in plaque regression. In this regard, risk factor modification was found to complement lipid-lowering medications to produce a slower rise in calcium scores.

In the end, accomplishment of regression may depend on both long-term lowering of LDL cholesterol (which prevents new deposits) and allowance of long-term exposure to the effects of HDL cholesterol (to remove plaques). Perhaps the answer depends on having a prolonged period of time to permit these processes to perform their action to the full.

Regarding the low incidence of adverse events in the group under study, this is understandable from the non-significant nature of the coronary disease affecting the whole study group. However it was only after the results of the study were analyzed that it became possible to be certain that relative lack of cardiac events can indeed be achievable by efficacious lipid-lowering therapy. In addition, the validity of such event-free development needed to be verified, and was double-checked by tracking patients' progress through clinical follow-up and telephone interviews. All individual results were further cross-checked by looking up death certificates held by the government registry.

A word of caution should be addressed to the direct evidence from epidemiologic studies that the organ doses corresponding to a common CT study (two or three scans, resulting in a dose in the range of 30 to 90 mSv) result in an increased risk of cancer [38]. Although the individual risk estimates are small, the concern about the risks from CT is related to the rapid increase in its use. On the basis of such risk estimates and data on CT use from 1991 through 1996, it has been estimated that 0.4% or more of all cancers in the United States may be attributable to the radiation from CT studies. A problem particularly arises for non-essential CT scans such as repeat scans ordered as the patient passes through the medical system, often simply because of a lack of communication. Thus CT scanning for documentation of CAD should be utilized only when indicated and targeted to achieving optimal management of cases with this problem. However, it should be stressed that in our study, the total dose of radiation from the electron beam CT scan, over a period of up to a decade, was well <3-5 mSv for any of the participants.

### Limitations of this study

There was no untreated control group for direct comparison in our study, and we therefore relied on the natural progression of calcium scores in other reported series for comparison.

The difficulties encountered in the follow-up of cases included firstly the inherent inter-scan variability of the calcium score, with possible confounding by varying treatment protocols due to changing ATP guidelines over the study period. Furthermore, knowledge of serial calcium score levels may have influenced the choice or aggressiveness of the treatment plan.

## Conclusions

Excluding cases with critical CAD (who were treated by revascularization and therefore did not form part of the cohort under investigation), this decade-long study of aggressive medical therapy in symptomatic CAD cases revealed that coronary plaques can be conveniently detected and followed by serial cardiac CT calcium scores. It confirmed our hypothesis that serial coronary calcium scanning can contribute towards disease documentation in a broad population, in addition to guiding decisions in patient management. Compared with an historical 33% rate of increase for untreated subjects, efficacious statin dosing aided by meaningful life-style modification in our study allowed 61.9% of cases to display a reduced rate of progression of calcified plaques (24% per year for the entire study population, contrasting with 16.4% per year on strict risk factor modification plus statin therapy), and a further 20.4% demonstrated actual regression of plaque burden as well. In addition, the rate of adverse events was extremely low (affecting only 4.4% of the study group) during the more than a decade of follow-up.

The encouraging results of this study are not only gratifying to the study subjects but also supported the findings of the COURAGE TRIAL, which should accordingly offer added confidence to physicians in adopting a non-invasive strategy as a viable option for managing CAD in properly selected patients.

## Competing interests

The authors declare that they have no competing interests, and that this study was not supported by any specific grant from any institution.

## Authors' contributions

VKG, CPL, SM, JAR, SA, MJB participated in contributions to conception, analysis and interpretation of data. VKG read all the CT examinations. SM revised the manuscript critically. MJB supervised and JAR, SA commented the study. All authors read and approved the final manuscript.
